# Potential Toxicity of Medicinal Plants Inventoried in Northeastern Morocco: An Ethnobotanical Approach

**DOI:** 10.3390/plants10061108

**Published:** 2021-05-31

**Authors:** Loubna Kharchoufa, Mohamed Bouhrim, Noureddine Bencheikh, Mohamed Addi, Christophe Hano, Hamza Mechchate, Mostafa Elachouri

**Affiliations:** 1 Laboratory of Bioresources, Biotechnology, Ethnopharmacology and Health, URAC-40, Department of Biology, Faculty of Sciences, Mohammed First University, Oujda 60040, Morocco; khloubna43@gmail.com (L.K.); mohamed.bouhrim@gmail.com (M.B.); bencheikh_noureddine1718@ump.ac.ma (N.B.); elachourimostafa@gmail.com (M.E.); 2 Laboratoire d’Amélioration des Productions Agricoles, Biotechnologie et Environnement, (LAPABE), Faculté des Sciences, Université Mohammed Premier, Oujda 60000, Morocco; m.addi@ump.ac.ma; 3 Laboratoire de Biologie des Ligneux et des Grandes Cultures, INRAE USC1328, Campus Eure et Loir, Orleans University, 45067 Orleans, France; christophe.hano@univ-orleans.fr; 4 Laboratory of Biotechnology, Environment, Agrifood and Health, Faculté des Sciences Dhar el Mahraz, University of Sidi Mohamed Ben Abdellah, Fez 30050, Morocco

**Keywords:** ethnobotany, traditional medicine, toxic plants, toxicity, Africa

## Abstract

Herbal medicine and its therapeutic applications are widely practiced in northeastern Morocco, and people are knowledgeable about it. Nonetheless, there is a significant knowledge gap regarding their safety. In this study, we reveal the toxic and potential toxic species used as medicines by people in northeastern Morocco in order to compile and document indigenous knowledge of those herbs. Structured and semi-structured interviews were used to collect data, and simple random sampling was used as a sampling technique. Based on this information, species were collected, identified, and herbarium sheets were created. The collected data were analyzed using two quantitative indices: informant consensus factor (ICF) and fidelity level (Fl), as the degree of these indices give an insight into the level of toxicity of a given plant. The results revealed the knowledge of 55 species belonging to 36 families. The most represented families were Apiaceae, Asteraceae, Solanaceae, and Fabaceae. Furthermore, the majority of the species cited were herbs (67%), and the most common toxic parts were seeds, followed by leaves and roots. According to the informant consensus factor, death (0.81%) had the highest agreement, followed by the urological (0.76%) and skin (0.75%) categories. The most significant plants in terms of fidelity level were *Solanum sodomaeum* L. and *Nerium oleander* L. for death, *Arisarum vulgare* O. Targ. Tozz., *Mentha spicata* L., and *Morus alba* L. for the digestive category, *Petroselinum crispum* (Mill.) Fuss. and *Citrus x aurantium* L. for cardiovascular category, *Urtica dioica* L. for skin category, *Datura stramonium* L, and *Ephedra altissima* Desf. for neurological category, and finally *Crocus sativus* L. for general and unspecified category. This work highlights a valuable traditional knowledge of poisonous and potential poisonous plants in northeastern Morocco. Further phytochemical and toxicological research is needed to determine the safety of these prized herbs.

## 1. Introduction

Over the last decade, there has been a great revival of reliance on herbal product medication to manage various ailments [1,2,3]. According to the World Health Organization (WHO), 80% of the world’s population, especially people in developing countries, is dependent on traditional medical practices for some aspect of primary health care [4]. In Morocco, the use of plants for medical care has been practiced since time immemorial and patients rely more on folk medicine: it is estimated that between 50% and 75% of the local population relies on traditional Moroccan remedies [5,6,7,8,9].

Like other regions in Morocco, the people of the northeastern region of the country are more concerned with the use of herbal medicine, and many of them have been collecting and preparing plants for medicinal purposes and the relief of disease symptoms [10,11]. However, the widespread belief that herbal drugs are perfectly safe and devoid of adverse effects is untrue and misleading [12,13]. Herbs can cause a wide range of undesirable or adverse reactions, some of which can result in severe injuries, life-threatening conditions, and even death [14]. Despite the popularity of these herbal products and their widespread use, the scientific evidence and assessment of the safety and toxicity of medicinal plants has been largely ignored, with the exception of a recent bibliographic work published by our team in which we documented a list of 89 toxic plants used traditionally by people living in this area [15]. In this regard, we conducted this fieldwork in order to assess local people’s knowledge of the toxicity of medicinal plants. To the best of our knowledge, the current study is the first to deal with the ethnotoxic knowledge of herbs. Throughout this work, we attempt to document and analyze traditional knowledge about toxic plants used as medicine by people in northeastern Morocco using both qualitative and quantitative approaches including various indexes such as informant consensus factor (ICF) and fidelity level (Fl).

## 2. Results and Discussion

### 2.1. Demographic Profile of the Informants

The demographic features of the informants were determined and recorded in this ethnobotanical survey (Table 1). The results revealed that 386 informants (345 were local inhabitants and 41 were herbalists) in the study zone belonged to three various northeastern Moroccan districts including 41.70% of Oujda Prefecture, 32.90% of Berkane Province, and 25.38% of Jerada Province. Of all interviewed persons, 67.61% were females, and 32.38% were males. The women’s responsibilities to care for all family members (husband and children) including nutrition and medication, and the fact that they were most often at home, could explain the high percentage observed (67.61%). These observations were consistent with several studies conducted in Morocco [6,7,10]. In terms of age, we found that out of the 386 participants, 56 persons (14.50%) were less than 30 years, 241 (62.43%) were between the ages of 30–60 years, 74 (19.17%) were between the ages of 60–80 years, and 15 (3.88%) were over the age of 80 years. Similar results have been reported in several studies [16,17]. Previous research has shown that the practice of phytotherapy is always more important for the elderly than for the young [18,19]. As a result, people of this age are more often responsible for the health of their families [20]. During the course of the study, it was observed that 43.00% of the participants were illiterate, 25.64% had completed primary school, 16.83% had completed university, and 14.50% had completed high school. These findings corroborate those obtained in various Moroccan ethnobotanical studies [21,22]. People with a lower educational level, in fact, have more expertise in the field of traditional medicine and medical folk botany [23]. Regarding the participants’ habitat, 60.10% were living in rural communities, and 39.10% were living in urban areas. These results are consistent with those previously reported in oriental Morocco [10]. Bellakhdar [24] indicates that rural participants are the primary consumers of medicinal plants because the rural suppliers maintained close contact with nature, which still provides them with many resources.

### 2.2. Floristic Characteristics of Poisonous Plants

**Figure 1:**
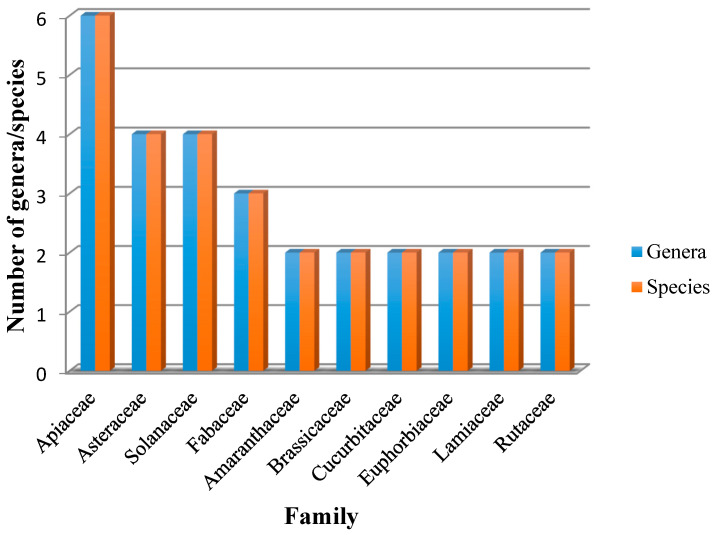
The main families of plants.

**Figure 2:**
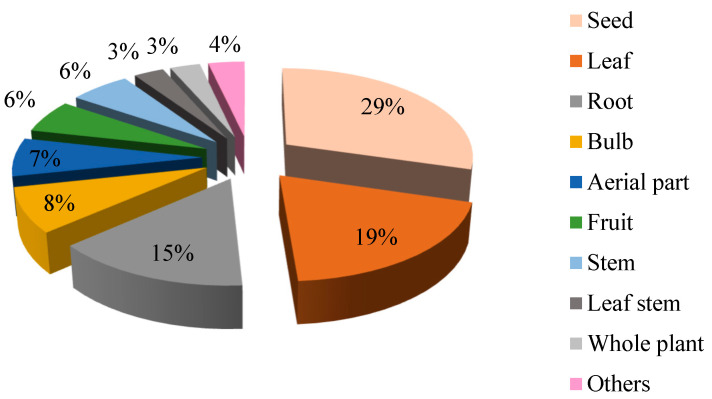
Percentage of the different parts.

The present ethnobotanical survey revealed 55 toxic and potential toxic herbs from 36 botanical families and 36 genera. The plant family with the highest number of species was Apiaceae, which contributed six species (11%), followed by the Solanaceae and Asteraceae families, which contributed four species each (7%), and the Fabaceae family, which contributed three species (5%) (Figure 1). Otherwise, the Amaranthaceae, Brassicaceae, Cucurbitaceae, Euphorbiaceae, Lamiaceae, and Rutaceae families each have two species (4%), and the remaining 26 families each have only one species (2%). Plant taxonomy at the family level is an important factor in determining the usefulness of plant species to local people. Some plant families are more useful than others in certain application categories [25]. The same logic applies to toxic and potential toxic species [26]. The Apiaceae, Asteraceae, Solanaceae, and Fabaceae families have been identified as the leading families of plants in our study area. This is consistent with our previous study in northeastern Morocco, where we identified 89 plants distributed across 45 families, the most dominant of which were Apiaceae, Fabaceae, Asteraceae, Brassicaceae, Lamiaceae, and Solanaceae [15]. A study conducted in India concluded that species belonging to the Fabaceae, Asteraceae, Apocynaceae, Solanaceae, and Euphorbiaceae families were frequently cited as toxic by local residents [17]. The toxicity of these families has been attributed to the presence of specific compounds such as acrid substances, alkaloids, heterosides, photosensitizing compounds, saponins, or volatile oils in Asteraceae; tropane, pyrrolidine, and pyrrolic alkaloids, proto- and glycol-alkaloids, or cardenolides in Solanaceae; and alkaloids, coumarin, heterosides, saponins, photosensitizing compounds, or selenium in Fabaceae [17,27]. Among the plants collected, herbs were the most common and represented 67% taxa, followed by shrubs (18% taxa) and trees (15% taxa). These results are consistent with previous studies [17]. Among the different toxic parts, seed toxicity was the highest (30%), followed by leaf toxicity (19%), root toxicity (14%), bulb toxicity (8%), aerial part toxicity (7%), fruit toxicity (6%), stem toxicity (6%), leaf stem toxicity (3%), and whole plant toxicity (3%) (Figure 2). These reports are in contrast with the previous study revealing the predominance of whole plant toxicity [28].

### 2.3. Traditional Knowledge of Poisonous Herbs

The documented poisonous and potentially poisonous plants and their ethnomedicinal data, along with a local name, life form, traditional uses, route of administration, poisonous part, and toxic signs, are summarized in Table 2. Our ethnobotanical investigation reached 386 participants in northeastern Morocco, and 148 persons responded. Percentage knowledge of poisonous and potential poisonous plants was 38.34% within the study population. A total of 55 medicinal plant species have been reported to be toxic or to have some toxicity. Twenty-six of these species were mentioned by fewer than three informants each. The remaining 29 species were mentioned by at least three different informants. Ethnobotanical information showed that all of the selected species have toxic effects such as neurological (dizziness, delirium, madness, hallucination, migraine, headache, sedation, loss of consciousness, sleep disturbance), digestive (vomiting, diarrhea, gastroenteritis, constipation, nausea, indigestion, stomachache, bloating, abdominal pain, mouth, bile problems, jaundice, gallbladder), skin (skin irritation, itchy, inflammation, skin problems), Urological (kidneys problems, diuretic, bladder diseases), cardiovascular (cardiac problems, hypertension, hypotension), and female genitals (abortion, sterility). Among the 55 species collected from the various northeastern Moroccan regions, *Nerium oleander* L., *Carlina gummifera* (L.) Less., *Cannabis sativa* L., *Peganum harmala* L., *Allium sativum* L., *Aristolochia fontanesii* Boiss. & Reut., *Trigonella foenum-graecum* L., *Glycine max* (L.) Merr., *Daphne gnidium* L., and *Ricinus communis* L. were the most cited plant species. According to the results presented in our previous study, *Nerium oleander* L., *Carlina gummifera* (L.) Less., *Aristolochia fontanesii* Boiss. & Reut., and *Ricinus communis* L. are all considered as severe poisonous plant species [15,29,30,31].

### 2.4. Quantitative Analysis of Data

To quantify our data, we used two ethnobotanical indexes adapted to toxicological studies: the informant consensus factor (ICF), which indicates the agreement about the toxicity of plant species, and the fidelity level (Fl), which indicates the most important species causing a particular illness.

#### 2.4.1. Informant Consensus Factor (ICF)

Based on the major toxicity effects, we classified the plant species listed in the present study into 12 groups using the International Classification of Primary Care-2^nd^ Edition (ICPC-2) [32]. Our finding indicated that the value of the informant consensus factor (ICF) ranged from 0 to 0.81 (Table 3). The elevated value was assigned to death (0.81), with 94 toxic citations for 18 herbs, followed by urological (ICF = 0.76; 14 toxic reports, four species), neurological (ICF = 0.73; 76 toxic reports, 21 species), digestive (ICF = 0.67; 95 toxic reports, 32 species), skin (ICF = 0.66; 10 toxic reports, four species), cardiovascular (ICF = 0.63; 31 toxic reports, 12 species), eye (ICF = 0.57; eight toxic reports, four species), female genitals (ICF = 0.47; 18 toxic reports, 10 species) and fever, allergy, pain general category with a ICF of 0.30 (11 toxic reports, eight species). The elevated value of ICF for some categories reflected a high level of homogeneity in consensus regarding the toxicity of plants. The high ICF for death may indicate a significant interrelationship between informants for a poisonous plant that causes this category. A low agreement observed between the informants was noted for plants affecting endocrine, metabolic, and nutritional category, respiratory category and blood, blood forming organs, and immune mechanism categories with ICF value zero for each one. According to Saleh Al-Qur’an [28], the low ICF value observed in our study could be attributed to a lack of communication among people in various areas.

#### 2.4.2. Fidelity Level (FL)

In Table 4, we regrouped the toxic plant species according to their corresponding fidelity level. Our results showed that the value of the fidelity level of plant species for a specific disease varied between 3.84 and 100%.

- Concerning the death category, the most important species were *Solanum sodomaeum* L. (Fl = 100%), *Nerium oleander* L. (Fl = 77.14%), and *Citrullus colocynthis* (L.) Schrad. (Fl = 66%).
- For the digestive category, *Mentha spicata* L. (Fl = 100%), *Arisarum vulgare* O. Targ. Tozz. (Fl = 100%), *Morus alba* L. (Fl = 100%), *Viscum album* L. (Fl = 100%), and *Ruta montana* (L.) L. (Fl = 80%).
- The most common plants in the cardiovascular category were *Petroselinum crispum* (Mill.) Fuss (Fl = 100%), *Citrus x aurantium* L. (Fl = 80%), and *Salvia officinalis* L. (Fl = 80%).
- For the neurological category, the plant with the highest Fl (100%) were *Ephedra altissima* Desf., *Papaver somniferum* L., *Datura stramonium* L., and *Anastatica hierochuntica* L.
- *Crocus sativus* L. was reported with Fl of 100% for the general and unspecified category.
- For the skin category, we found *Urtica dioica* L. (Fl = 87.5%) was the most important.
- For the female genital category, *Glycine max* (L.) Merr. (Fl = 50%) was the most important.

The toxic species with high fidelity level constitute the most important toxic herbs cited by the informants, causing a particular illness category. Furthermore, the plants causing repetitive are more likely to have some toxic compounds. For the plants with low fidelity level, we can note the following species:

- *Peganum harmala* L. and *Cannabis sativa* L. for the digestive category;
- *Nerium oleander* L. for the cardiovascular and eye categories;
- *Glycine max* (L.) Merr. for the neurological category;
- *Peganum harmala* L. for the female genital category;
- *Allium sativum* L. for the skin category;
- *Carlina gummifera* (L.) Less. and *Cannabis sativa* L. for the respiratory category; and
- *Trigonella foenum-graecum* L. for endocrine, metabolic and nutritional, blood, blood forming organs, and immune mechanism.

These plants may cause little harm. Furthermore, the low ICF value observed in our study could be attributed to a lack of communication among people working in various areas of study as previously hypothesized [17].

## 3. Materials and Methods

### 3.1. Study Area

**Figure 3:**
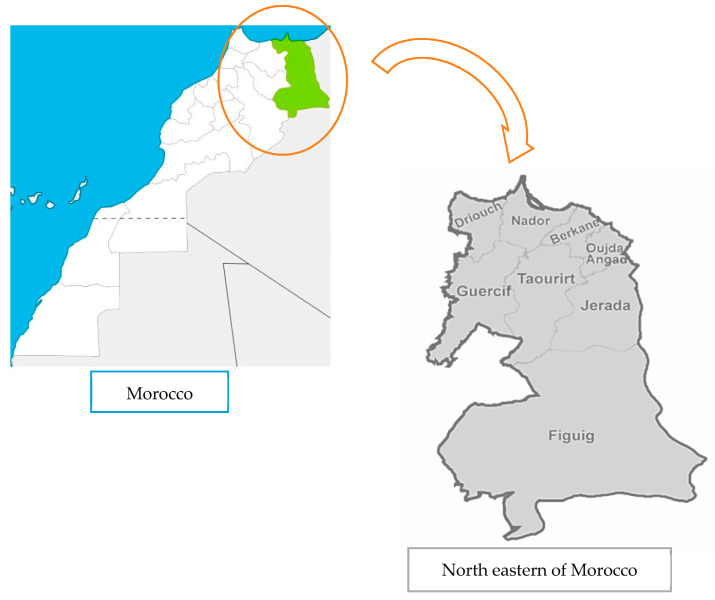
Geographical position of northeastern Morocco.

The study zone is located in the northeastern region of Morocco (Figure 3). This part of the country is one of the twelve regions of Morocco including eight province districts with a total of 90,130 km^2^ (12% of the national territory). According to the national census report published in 2014, the population in this region reached 2,314,346 (6.8% of the national population) with a density of 26 persons per square kilometer [33]. The dominant language in this region was the dialect Arabic. In the second position, we found the Berber language, which is subdivided into two dialects, with low proportions: *Tarifit* in the north of the region and *Tashelhit* in the south. The climatic variability in the region extends from the north to the Saharan in the south. Because of these characteristics, the region has a high level of biodiversity including a diverse flora and, in particular, medicinal plants.

### 3.2. Ethnobotanical Data Collection

From April 2017 to June 2019, a fieldwork survey was carried out in order to lay down a thorough ethnobotanical study of poisonous and potential poisonous species with significant toxicity effects. Ethnobotanical data were collected from 386 informants aged 20 and up from various districts in northeastern Morocco (Table 1). The study area included the Oujda-Angad, Berkane, and Jerada districts. These areas were selected because of their cultural and botanical diversities. The sampling technique used in this ethnobotanical investigation was simple random sampling. The data were obtained through structured and semi-structured interviews and face-to-face conversations with local inhabitants and herbalists of the study areas. Permission were obtained from the interviewees prior to the interview. The questionnaires were divided into two sections: the first focused on informant profiles such as location, age, gender, and academic level, while the second collected ethnobotanical data about the plants including local names, traditional uses, administration route, toxic part, and toxic signs. Only plants that were mentioned more than once were selected for analysis.

### 3.3. Plant Collection and Identification

The names of the plant species mentioned by the interviewees were identified using technical documentation related to Moroccan flora [34,35,36] and compared with the specimens already in the Herbarium of Mohammed First University Oujda-Morocco (HUMPOM). A voucher number was assigned to each specimen and deposited in the HUMPOM. The scientific names of plants selected were checked against The Plant List Database [37].

### 3.4. Analysis of Ethnomedicinal Data

The data recorded on the questionnaire sheets during the survey trips were sorted in an Excel 2016 sheet and analyzed for quantitative indices like the informant consensus factor (ICF) and fidelity level (Fl).

#### 3.4.1. Informant Consensus Factor (ICF)

The informant consensus factor (ICF) was initially used to determine whether respondents agreed on the use of plants to treat various ailments. This parameter was used in this study, with minor modifications, to determine the informants’ agreement on the toxicity of selected species. 

The ICF was calculated using the following formula [38]:

ICF = Nur −Nt/Nur − 1, where Nur refers to the number of reports for a particular ailment category, and Nt refers to the number of plants affected by this particular ailment category. This index can only have a value between 0 and 1. A high value (close to 1) indicated that the toxicity of the revealed plants is primarily known by the informants and/or how the toxicity of the plants is exchanged between respondents. A low value of this parameter, on the other hand, indicated that the informants disagreed on the toxicity of specific plants or that the respondents did not exchange their knowledge about the toxicity [39,40,41].

#### 3.4.2. Fidelity level (FL)

The fidelity level (Fl) of each plant was calculated as commonly adopted [42]:

Fl (%) = (Ns/N) × 100, where Ns is the number of respondents who informed the toxicity of a specific plant for specific toxicity, and N is the total number of informants who informed all significant toxicities of the plant. The plants mentioned once were excluded from this analysis.

## 4. Conclusions

In total, fifty-five medicinal plants belonging to 36 families were here reported to be toxic or present a potential toxicity by indigenous people from northeastern Morocco. These results indicated that this population has extensive traditional knowledge of medicinal plants and their harmful effects, and quantitative analyses showed that the inventoried plants may negatively impact different organ and pose a risk to human health. Despite the fact that some of the reported plants as used commonly in culinary and acute health problems, the dose is a key factor defining where the effect tends to be therapeutic or toxic. Caution should be exercised when using these plants, particularly for medicinal purposes, and adequate information on these plants including toxicity, composition, and safe doses should be obtained. In the present study, the FI and ICF results could be followed to presume the toxicity (high FI and ICF) or the potential toxicity (low FI and ICF). In accordance with this, it is suggested that additional studies be conducted (i) to confirm traditional information associated with poisonous plants using appropriate experiments, and (ii) to determine the identity of toxic phytochemicals associated with poisonous plants.

## Figures and Tables

**Table 1:** Informant socio-demographic characteristics.

Socio-Demographic Variables	Sample	
	Number	Percentage (%)
**District**
Oujda	161	41.70
Berkane	137	32.90
Jerada	88	25.38
**Function**
Ordinary inhabitant	345	89.37
Herbalists	41	10.63
**Habitat**
Urban	154	39.89
Rural	232	60.10
**Age (Years)**
<30	56	14.50
30–60	241	62.43
60–80	74	19.17
>80	15	3.88
**Gender**
Male	125	32.38
Female	261	67.61
**Study Level**
Illiterate	166	43
Primary	99	25.64
High school	56	14.50
University	65	16.83

**Table 2:** List of toxic plants cited toxic by people in northeastern Morocco.

Family Scientific Name	Local Name	Herbaceous Life Form/Voucher Number	Rout of Administration	Traditional Uses	Poisonous Part	Toxic Signs	NC
**Amaranthaceae**							
*Dysphania* *ambrosioides* (L.) Mosyakin & Clemants	Mkhinza	Herb/HUMPOM571	Internal and external uses	Headache, migraine, fever	Leaf Aerial part	Madness, dizziness, death (oral), gallbladder toxicity	7
*Haloxylon scoparium* Pomel	Ramt	Herb/HUMPOM572	Internal uses	Diabetes, scorpion bite	Aerial part	Vomiting, abdominal pain, pain general	3
**Amarillydaceae**							
*Allium sativum* L.	Thouma	Herb/HUMPOM573		Food, cold, hypertension, asthma, cough, dental pain, ear pain, digestive, antibiotic, weight loss, hair strengthening, immunity, eczema, influenza, hemorrhoids, gum disease	Bulb	Hypotension, stomach-ache, allergy, nephrotoxicity, stomach burning	22
**Anacardiaceae**							
*Pistacia lentiscus* L.	Dro/meska	Tree/HUMPOM574	Internal uses	Uterus cancer, diabetes, gastro-intestinal disorders	Leaf, fruit	Constipation, skin irritation	2
**Apiaceae**							
*Conium maculatum* L.	Ziyyata	Herb/HUMPOM575	Internal uses	Cold	Fruit, seed	Neurological problems, abdominal pain, vomiting	4
*Coriandrum sativum* L.	Kosbare, Kessbour	Herb/HUM-POM576	Internal and external uses	Food, mouth ulcers, frequent urination	Leaf, seeds, leafy stem	Forgetting, sleep, dizziness, neurological problems, allergy	5
*Ferula communis* L.	Kelkha L-klekh Fasoukh	Herb/HUMPOM577	Internal and external uses	Cold, food, magic, anthelmintic, female sterility.	Stem, resin	Abortion, irritation, death, pain general, neurological toxicity	7
*Foeniculum vulgare* Mill.	Bassbass	Herb/HUMPOM578	Internal and external uses	Abdominal pain, intestine, maternal milk.	Seed	Dizziness, hypotension, facial pills	3
*Petroselinum crispum* (Mill.) Fuss	Maâdnousse	Herb/HUMPOM579	Internal and external uses	Food, hypertension, hair	Leaf	Hypotension	2
*Thapsia garganica* L.	Deryas	Herb/HUMPOM580	Internal uses	Antispasmodic, rheumatism, women sterility, cough	Root	Abortion (animal), vomiting and diarrhea	4
**Apocynaceae**							
*Nerium oleander* L.	Dafla	Shrub/HUMPOM581	External uses	Stop nose bleeds, tonsillitis, swollen gums, cold, headache, teeth, rheumatism, sedative, mumps, influenza, jaundice, ear infections, mouth-ulcers, diabetes, torticollis, irritable bowel syndrome, skin problems	Whole plant	Death (oral), increased heart rate and irregularity, toxic to eyes, abdominal, pain, stomachache, digestive system disorders	35
**Araceae**							
*Arisarum vulgare* O. Targ. Tozz.	Tabgouga	Herb/HUMPOM582	External and internal uses	Emetic, purgative, dermatological problems	Tuber	Mouth inflammation, intestinal pain, vomiting, hepatotoxicity	2
**Araliaceae**							
*Hedera helix* L.	Louwaya	Shrub/HUMPOM583	External uses	Rheumatism, skin abscesses	Leaf, fruit	Death (oral)	2
**Aristolochiaceae**							
*Aristolochia* *fontanesii* Boiss. & Reut.	Baraztem	Shrub/HUMPOM584	External and internal uses	Cancer, skin infection, intestinal parasites, diabetes	Root, whole plant	Nephrotoxicity, hepatotoxicity, vomiting, dizziness, indigestion, death	15
**Asparagaceae**							
*Drimia maritima* (L.) Stearn	Bes’al ed-dib Bassila	Herb/HUMPOM585	External and internal uses	Detoxification, dental pain, abdominal pain	Bulb	Diarrhea, death, dizziness, nausea, vomiting	6
**Asteraceae**							
*Carlina* *gummifera* (L.) Less.	Addad	Herb/HUMPOM586	External and internal uses	Headache, respiratory system, sterility, rheumatism, skin diseases, mouth pain, freckles on the face, whiten the teeth, facilitates childbirth, hair loss.	Root	Vomiting, diarrhea, swilling, death, digestive disorders	25
*Warionia saharae* Benth. & Coss.	Affessass	Herb/HUMPOM587	External and internal uses	Softens and strengthens hair, Gastroenteritis	Leafy stem	Nephrotoxicity	2
*Artemisia arborescens* (Vaill.) L.	Chiba	Herb/HUMPOM588	External and internal uses	Hypoglycemic, otitis	Aerial part	Diarrhea, dizziness, convulsion	2
*Launaea arborescens* (Batt.) Murb.	Mmû-lbeyna	Shrub HUMPOM589	Internal uses	Hypoglycemic, respiratory problems	Latex	Death	2
**Boraginaceae**							
*Borago officinalis* L.	Lsan tawr El hricha	Herb/HUMPOM590	External uses	Knee pains	Root	Death (oral)	2
**Brassicaceae**							
*Anastatica hierochuntica* L.	Lkmicha, Keff-maryam, Tamkelt	Herb/HUMPOM591	Internal uses	Strengthen the uterus	Leafy stem	Neurological disorders, sleep disturbance	2
**Brassicaceae**							
*Lepidium sativum* L.	Hab rachad	Herb/HUMPOM592	Internal and external uses	Hair, anemia, cough, intestinal gas, strengthen hair, regulates the menstrual cycle, maternal milk	Seed	Indigestion, diuresis, abortion, dizziness hepatotoxicity, hypertension	8
**Cannabaceae**							
*Cannabis sativa* L.	Lkif	Herb/HUMPOM593	External uses	Hair loss, hair lengthening and thickening, against dandruff, strengthen hair, calming	Leaf, seed	Ecstasy, hepatotoxicity madness, sedation, neurological toxicity, respiratory problems, dizziness, lose consciousness, neurological toxicity, death	26
**Caryophyllaceae**							
*Herniaria cinerea* DC.	Harasst lahjar	Herb/HUMPOM594	Internal uses	Kidney stones	Aerial part	Hypertension	2
**Cucurbitaceae**							
*Bryonia cretica* subsp. *dioica* (Jacq.) Tutin	Aneb dib	Herb/HUMPOM595	Internal and external uses	Hair loss, urinary tract infection	Whole plant	Harmful to pregnant woman, toxic for people with stomach diseases	2
*Citrullus colocynthis* (L.) Schrad.	H’adej Handa’al	Herb/HUMPOM596	Internal and external uses	Cancer, diabetes, detoxification, diuretic	Fruit	Death (high doses), abortion, diarrhea vomiting	9
**Cupressaceae**							
*Tetraclinis articulata* (Vahl) Mast.	Ar-âr	Tree/HUMPOM597	Internal and external uses	Colds, headache, skin diseases	Leaf	Neurotoxicity	2
**Ephedraceae**							
*Ephedra altissima* Desf.	Laâlenda	Shrub/HUMPOM598	Internal uses	Cancer	Aerial part	Dizziness	3
**Euphorbiaceae**							
*Euphorbia resinifera* O. Berg	Takiwt Takawt	Shrub/HUMPOM599	Internal and external uses	Hair care, diabetes	Seed	Death	2
**Fabaceae**							
*Glycine max* (L.) Merr.	Soja	Herb/HUMPOM600 Shrub/	Internal uses	To weight gain, against menopause, appetite increases female hormones, hypertension colon	Seed	Sterility, sedation, sleep, diarrhea, blocks absorption of nutrients (high dose), colic, nausea, constipation, hypertension	2
*Retama monosperma* (L.) Boiss.	Rtem	HUMPOM601	External uses	Magic, hypoglycemia	Stem	Death (high doses), abortion	12
*Trigonella foenum-graecum* L.	Lhelba	Herb/HUMPOM602	Internal uses	Anemia, appetite, weight gain, blood detoxification, digestive system, facilitate food absorption, cold, antiparasitic	Seeds	Jaundice, harmful to pregnant woman, renal pain, hypertension, gastrointestinal diseases, nausea, decreases immunity, stomachache (in high doses), abortion	12
**Gentianaceae**							
*Centaurium* *erythraea* Rafn.	Gosset l-hayya, Marraret al-hench	Herb/HUMPOM603	Internal uses	Infertility, skin diseases	Seed	Nausea and dizziness	2
**Hypericaceae**							
*Hypericum perforatum* L.	Hachicht lkalb	Herb/HUMPOM604	Internal and external uses	Asthma, varicose veins	Flower	Death, nephrotoxicity, dermatological problems	4
**Iridaceae**							
*Crocus sativus* L.	Za’fran lhor	Herb/HUMPOM605	Internal uses	Digestion, anemia, asthma, cough	Stigma	Hypotension, dry mouth, allergic reaction (flushing of the face)	2
**Lamiaceae**							
*Mentha spicata* L.	Naana’a	Herb/HUMPOM606	Internal and external uses	Stomachache, lose weight, heart problems, cosmetic	Leaf Aerial part	Intestinal gas, colon bloating	8
*Salvia officinalis* L.	Salmia	Shrub/HUMPOM607	Internal	Diabetes, balance female hormones, painful menstrual periods, influenza, pain killer, digestive system	Aerial part Leaf	Fever, nausea	5
**Molluginaceae**							
*Corrigiola litoralis* L.	Serghina	Herb/HUMPOM608	External uses	Cosmetic	Root Leaf	Diarrhea (oral), abdominal pain	2
**Moraceae**							
*Morus alba* L.	Toute	Tree/HUMPOM609	Internal uses	Anemia, cough	Fruit	Mouth inflammation (high doses)	2
**Myristicaceae**							
*Myristica fragrans* Houtt	Lgouza	Tree/HUMPOM610	Internal uses	Food	Fruit	Dizziness	2
**Nitrariaceae**							
*Peganum harmala* L.	Harmel	Herb/HUMPOM611	Internal and external uses	Headache, cold, hair care, constipation, fever, anorexia, diarrhea, abortion, lengthening hair, arthritis, rheumatism, diabetes, magic, bad spirit, facilitation of childbirth	Seed Aerial part Leaf	Neurological disorders vomiting, abortion hepatoxicity, eye irritations, death, dizziness	23
**Solanaceae**							
*Solanum sodomaeum* L.	Matichat lahmir	Shrub/HUMPOM612	External uses	Rheumatism	Berries	Death	2
**Papaveraceae**							
*Papaver somniferum* L.	Kherkhacha	Herb/HUMPOM613	Internal uses	Calming, sedative	Fruit	Neurological disorders	6
**Ranunculaceae**							
*Nigella sativa* L.	Sanouj	Herb/HUMPOM614	Internal uses	Respiratory infections, cold, diabetes, digestive disease, muscle stiffness	Seed	Dizziness (high doses), irritations, hepatotoxicity	5
**Rhamnaceae**							
*Ziziphus lotus* (L.) Lam.	Sedr-Nbeg	Shrub/HUMPOM615	Internal uses	Digestive disease, diabetes	Root	Bladder toxicity	2
**Rosaceae**							
*Prunus dulcis* (Mill.) D.A.Webb	Louz lmor	Tree/HUMPOM616	Internal and external uses	Hypertension, diabetes, fever, hair care	Seed Fruit	Hypotension (>3 seeds), fever, death (high doses)	5
**Rutaceae**							
*Citrus x aurantium* L.	Ranje	Tree/HUMPOM617	Internal uses	Hypertension, fever, cod, diarrhea, cosmetic, increase blood flow and circulation	Fruit Leaf pulp	Hypoglycemia, abortion, cardiac problem, hypertension	5
*Ruta montana* (L.) L.	Fijel	Herb/HUMPOM618	Internal and external uses	Diarrhea, headache, fever, menstrual disorders, diabetes, bad spirit	Whole plant Leaf Aerial part	Diarrhea, vomiting Digestive disorders, nervous disorder	5
**Santalaceae**							
*Viscum album* L.	Lenjbar	Tree/HUMPOM619	Internal uses	Diarrhea, throat disorders	Seed	Constipation Hepatotoxicity	2
**Solanaceae**							
*Atropa* *Belladonna* L.	Zbib lidur	Herb/HUMPOM620	Internal uses	Stimulant and aphrodisiac,	Berries seed	Nervous disorder, colic, gastroenteritis, death	5
*Datura stramonium* L.	Chdeq jmel	Herb/HUMPOM621	Internal uses	Headache, menstrual blood, sexual stimulant, sedative	Seed	Delirium, madness, hallucination	4
*Hyoscyamus albus* L.	Bounarjouf	Herb/HUMPOM622	Internal and external uses	Skin infections, eczema, sedative	Seed Aerial part	Dizziness, hallucination	2
**Thymelaeaceae**							
*Daphne gnidium* L.	Lezzâz	Herb/HUMPOM623	external uses	Hair hydration, hair elongation	Leaf Aerial part	Dizziness, fainting eye irritation, skin problems	10
**Euphorbiaceae**							
*Ricinus communis* L.	Kherouaa	Tree/HUMPOM624	Internal and external uses	Abdominal pain, constipation, cosmetic (hair and face), detoxification, eyelash strengthening	Seed (oil)	Vomiting, eye pain, death, nausea	10
*Urtica dioica* L.	Lhoriga	Herb/HUMPOM625	Internal and external uses	Menstrual pain, respiratory system diabetes, rheumatism, cold, kidneys	Aerial part Leaf	skin irritation, allergy, stomachache, skin problems	8

NC: Number of Citation.

**Table 3:** Category of illness and their informant consensus factor (ICF).

Ailments Categories	Ailments	Number of Reports	Number of Species	Informant Consensus Factor (ICF)
Death	Death	94	18	0.81
Urological	Kidneys problems, diuretic, bladder diseases	14	4	0.76
Neurological	Dizziness, delirium, madness, hallucination, migraine, headache, neurotoxicity, forgetting, sedation, lose consciousness, sleep disturbance, tonic-clonic seizures	76	21	0.73
Digestive	Vomiting, diarrhea, gastroenteritis, constipation, nausea, indigestion, stomachache, bloating, intestinal gas, absorption of nutrients, abdominal pain, mouth, liver problems, bile problems, jaundice, gallbladder	95	32	0.67
Skin	Skin irritation, itchy, inflammation, skin problems	10	4	0.66
Cardiovascular	Cardiac problems, hypertension, hypotension	31	12	0.63
Eye	Eye pain, eye irritation	8	4	0.57
Female Genital	Abortion, sterility	18	10	0.47
General and Unspecified	Fever, allergy, pain general	11	8	0.30
Endocrine, Metabolic and Nutritional	Hypoglycemia	1	1	0
Respiratory	Breathing problems	2	2	0
Blood, Blood Forming Organs and Immune Mechanism	Immune	1	1	0

**Table 4:** The plants with fidelity level values.

Category of Illness	Name of Species	Fidelity Level (Fl %)
**Death**	*Solanum sodomaeum* L.	100.00%
	*Nerium oleander* L.	77.14%
	*Citrullus colocynthis* (L.) Schrad.	66.00%
	*Carlina gummifera* (L.) Less.	64.00%
	*Prunus dulcis* (Mill.) D.A.Webb	60.00%
	*Ferula communis* L.	57.14%
	*Aristolochia fontanesii* Boiss. & Reut.	40.00%
	*Cannabis sativa* L.	34.61%
	*Drimia maritima* (L.) Stearn	33.33%
	*Hypericum perforatum* L.	20.00%
	*Peganum harmala* L.	17.39%
**Digestive**	*Mentha spicata* L.	100.00%
	*Arisarum vulgare* O. Targ. Tozz.	100.00%
	*Morus alba* L.	100.00%
	*Viscum album* L.	100.00%
	*Ruta montana* (L.) L.	80.00%
	*Thapsia garganica* L.	75.00%
	*Haloxylon scoparium* Pomel	66.66%
	*Artemisia arborescens* (Vaill.) L.	50.00%
	*Bryonia cretica* subsp. *dioica* (Jacq.) Tutin	50.00%
	*Pistacia lentiscus* L.	50.00%
	*Ricinus communis* L.	50.00%
	*Drimia maritima* (L.) Stearn	50.00%
	*Carlina gummifera* (L.) Less.	44.00%
	*Glycine max* (L.) Merr.	41.66%
	*Citrullus colocynthis* (L.) Schrad.	33.00%
	*Allium sativum* L.	31.18%
	*Conium maculatum* L.	25.00%
	*Trigonella foenum-graecum* L.	25.00%
	*Aristolochia fontanesii* Boiss. & Reut.	20.00%
	*Salvia officinalis* L.	20.00%
	*Nigella sativa* L.	20.00%
	*Nerium oleander* L.	17.14%
	*Lepidium sativum* L.	12.50%
	*Urtica dioica* L.	12.50%
	*Peganum harmala* L.	8.69%
	*Cannabis sativa* L.	7.69%
**Cardiovascular**	*Petroselinum crispum* (Mill.) Fuss	100.00%
	*Citrus x aurantium* L.	80.00%
	*Salvia officinalis* L.	80.00%
	*Allium sativum* L.	50.00%
	*Nigella sativa* L.	20.00%
	*Prunus dulcis* (Mill.) D.A.Webb	20.00%
	*Trigonella foenum-graecum* L.	16.66%
	*Lepidium sativum* L.	12.50%
	*Nerium oleander* L.	2.85%
**Neurological**	*Ephedra altissima* Desf.	100.00%
	*Papaver somniferum* L.	100.00%
	*Datura stramonium* L.	100.00%
	*Anastatica hierochuntica* L.	100.00%
	*Coriandrum sativum* L.	80.00%
	*Conium maculatum* L.	75.00%
	*Peganum harmala* L.	69.56%
	*Cannabis sativa* L.	61.53%
	*Daphne gnidium* L.	60.00%
	*Nigella sativa* L.	60.00%
	*Artemisia arborescens* (Vaill.) L.	50.00%
	*Lepidium sativum* L.	37.00%
	*Ruta montana* (L.) L.	20.00%
	*Drimia maritima* (L.) Stearn	16.66%
	*Ferula communis* L.	28.57%
	*Glycine max* (L.) Merr.	8.33%
**Female Genitals**	*Glycine max* (L.) Merr.	50.00%
	*Citrus x aurantium* L.	40.00%
	*Thapsia garganica* L.	25.00%
	*Trigonella foenum-graecum* L.	25.00%
	*Bryonia cretica* subsp. *dioica* (Jacq.) Tutin	20.00%
	*Ferula communis* L.	14.28%
	*Lepidium sativum* L.	12.50%
	*Citrullus colocynthis* (L.) Schrad.	11.00%
	*Peganum harmala* L.	8.69%
**Urological**	*Aristolochia fontanesii* Boiss. & Reut.	46.00%
	*Hypericum perforatum* L.	25.00%
	*Trigonella foenum-graecum* L.	16.66%
	*Lepidium sativum* L.	12.50%
	*Allium sativum* L.	4.54%
**Skin**	*Urtica dioica* L.	87.50%
	*Pistacia lentiscus* L.	50.00%
	*Daphne gnidium* L.	10.00%
**General and Unspecified**	*Crocus sativus* L.	100.00%
	*Haloxylon scoparium* Pomel	33.33%
	*Coriandrum sativum* L.	20.00%
	*Salvia officinalis* L.	20.00%
	*Prunus dulcis* (Mill.) D.A.Webb	20.00%
	*Ferula communis* L.	14.28%
	*Allium sativum* L.	13.63%
**Eye**	*Daphne gnidium* L.	30.00%
	*Ricinus communis* L.	20.00%
	*Nerium oleander* L.	2.85%
**Respiratory**	*Carlina gummifera* (L.) Less.	4.00%
	*Cannabis sativa* L.	3.84%
**Endocrine, Metabolic, and Nutritional**	*Citrus x aurantium L.*	20.00%
	*Trigonella foenum-graecum* L.	8.33%
**Blood, Blood Forming** **Organs, and Immune** **Mechanism**	*Trigonella foenum-graecum* L.	8.33%

## Data Availability

Data are available from the authors upon reasonable request.
